# The association between childbirth, breastfeeding, and uterine fibroids: an observational study

**DOI:** 10.1038/s41598-019-46513-0

**Published:** 2019-07-12

**Authors:** Giovanni Delli Carpini, Stefano Morini, Maria Papiccio, Matteo Serri, Valentina Damiani, Camilla Grelloni, Nicolò Clemente, Andrea Ciavattini

**Affiliations:** Woman’s Health Sciences Department, Università Politecnica delle Marche, Via F. Corridoni, 11, 60123 Ancona, Italy

**Keywords:** Ultrasonography, Reproductive signs and symptoms

## Abstract

The aim of this study was to investigate the effect of childbirth and breastfeeding on uterine fibroids and to identify the factors associated with size variations. This was a monocenter observational study carried on women with a sonographic diagnosis of uterine fibroids from January 2007 to December 2016, with no indication for immediate treatment, and who became pregnant within one year from diagnosis. All patients were re-evaluated six months after delivery. Fibroid diameters were compared between pre-pregnancy period, first, second, third trimester and post-delivery. The rate of “regressed” (growth of diameter <−40%), “unchanged” (growth of diameter between −40% and +40%) or “increased” (growth of diameter >+40%) fibroids at the post-delivery evaluation with respect to the pre-pregnancy state was calculated. One-hundred fifty-seven women were included in the final analysis. At the post-delivery ultrasound, a significant reduction of the fibroid diameter with respect to all previous examinations was observed, and there was no evidence of 67 (37.2%) fibroids. Ongoing breastfeeding was positively associated with an “unchanged” or “regressed” fibroid diameter (adOR 3.23, 95%CI: 1.35–7.70, p < 0.01). Smaller pre-gravidic fibroids were more likely to return to pre-pregnancy dimensions or to regress, with a cut-off of 32 mm for lactating women and of 26 mm for non-lactating women. In conclusion, fibroids seem to return to pre-pregnancy dimensions or to regress in the post-partum period. This process may be sustained by uterine involution and hormonal variations, with an additional role of breastfeeding.

## Introduction

Uterine fibroids represent the most common gynecological benign neoplasm in the reproductive years, and their incidence is directly related to age, ranging from 40–60% at 35 years old to 70–80% at 50 years old^1^. The prevalence of uterine fibroids in pregnant women vary from 0.1 to 10.7%^2–4^ and is probably underestimated, in consideration of the lower diagnostic accuracy of sonography during pregnancy and of the limitations of clinical diagnosis by physical examination in pregnant women. Given the association of uterine fibroids with age, their incidence during pregnancy will probably rise in the coming years, because of the current increasing trend of delaying child-bearing.

Even if an inverse association between fibroid risk and parity is well documented^5^, the effect of pregnancy on uterine fibroids is not yet fully understood; it is reported in the literature that fibroids may increase in size during early pregnancy and undergo to a stabilization or regression in the second and third trimester^6–10^. However, the global effect of pregnancy, including childbirth and post-partum, must be better clarified.

Considering the current indications for treatment of uterine fibroids that are (1) the presence of fibroid-related symptoms impacting the quality of life; (2) sub-mucosal fibroids in asymptomatic women with desire of pregnancy (in which case hysteroscopy represent the actual gold standard for diagnosis and treatment);^11,12^ (3) intramural fibroids distorting the uterine cavity in infertile patients according to their dimensions^13^, women which do not fall in these indications and who become pregnant need to be adequately managed in the light of the risk of adverse obstetric outcomes related to the size of fibroids which could grow during pregnancy^14^, of fibroid-related complication (torsion of pedunculated fibroid or necrosis with inflammatory peritoneal reaction)^15^, and also considering the risk of onset of fibroid-related symptoms and need of medical or surgical therapy if the increase in dimensions is maintained after childbirth^16^.

To this end, this observational study was performed on women with pre-pregnancy identification of uterine fibroids who spontaneously become pregnant, with the aim of monitoring fibroid size from early pregnancy up to six months after delivery and identify the factors that could influence the dimensional variations between pre-pregnancy and post-partum, with specific interest in pre-pregnancy dimensions of the fibroids and breastfeeding.

## Results

**Figure 1:**
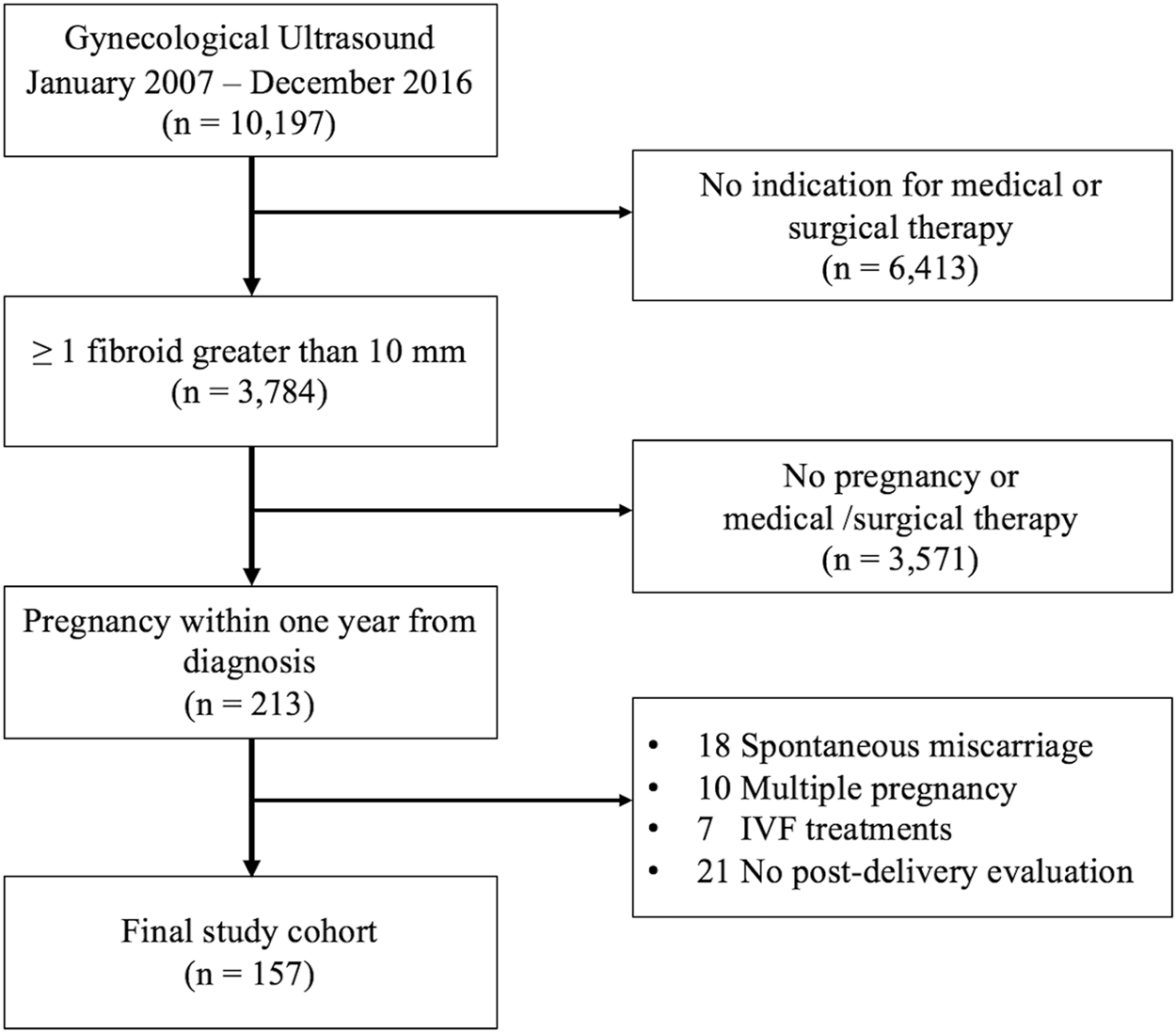
Study flow diagram.

Within the study period, 10,197 child-bearing age women underwent a gynecological ultrasound at our institution for mild gynecological symptoms, and 3,784 (37.1%) presented at least one uterine fibroid with a diameter greater than 10 mm. Among these, 3,571 (94.4%) did not present any indication for medical or surgical therapy, and 213 patients (5.6%) became pregnant within one year from diagnosis. Fifty-six of them (26.3%) did not meet the study inclusion and exclusion criteria (18 because of miscarriage, 10 had a multiple pregnancy, seven underwent *in-vitro* fertilization treatments, and 21 did not attend the ultrasound evaluation six months after delivery). Therefore, the remaining 157 women constituted the study cohort. Figure 1 reports the complete study flow diagram.

The mean ± standard deviation (SD) maternal age was 34.4 ± 4.8 years, and the mean ± standard deviation (SD) body mass index (BMI) was 24.6 ± 3.7 kg/m^2^. Fifty-seven (36.6%) women were nulliparous, and the median (interquartile range, IQR) number of previous pregnancies was 1 (0–1). Tobacco use in pregnancy was found in eight (5.1%) women. The mean ± SD gestational age at delivery was 38.9 ± 1.7 gestational weeks, and 15 (9.5%) women had a preterm birth. A caesarean section was performed in 45 (28.7%) patients. At the six-month post-delivery evaluation, 106 (67.5%) women were still breastfeeding.

At the enrollment pre-pregnancy visit, 119 patients (66.1%) presented a single fibroid and 11 (6.1%) presented two fibroids, nine (5%) women presented three fibroids, and three (1.7%) women presented four fibroids for a total number of fibroids of 180. The number, site, and localization of the identified fibroids remained constant from pre-pregnancy ultrasound until the third-trimester ultrasound, allowing the paired comparison between each fibroid. Table 1 reports the sonographic characteristics of fibroids identified during the five ultrasounds examinations.

**Table 1:** Sonographic characteristics of the identified fibroids during the five ultrasounds.

Characteristic	PPU (n = 180)	FTU (n = 180)	STU (n = 180)	TTU (n = 180)	PDU (n = 113)	p
Diameter (mm)	22 (15–30)	35 (28–43)	36 (30–42)	37 (30–43)	14 (0–30)	<0.001^a^
**Location**						
Anterior	62 (34.4)	62 (34.4)	62 (34.4)	62 (34.4)	32 (28.3)	1.000^b^
Posterior	94 (52.3)	94 (52.3)	94 (52.3)	94 (52.3)	62 (54.9)	
Right Lateral	6 (3.3)	6 (3.3)	6 (3.3)	6 (3.3)	5 (4.4)	
Left Lateral	9 (5.0)	9 (5.0)	9 (5.0)	9 (5.0)	7 (6.2)	
Fundal	9 (5.0)	9 (5.0)	9 (5.0)	9 (5.0)	7 (6.2)	
**Site**						
Subserosal	26 (14.5)	26 (14.5)	26 (14.5)	26 (14.5)	17 (15.0)	0.999^b^
Intramural	154 (85.5)	154 (85.5)	154 (85.5)	154 (85.5)	96 (85.0)	

PPU = pre-pregnancy ultrasound; FTU = first-trimester ultrasound; STU = second-trimester ultrasound; TTU = third-trimester ultrasound; PDU = post-delivery ultrasound Data are reported as median (IQR) or n (%) as appropriate.

^a^Kruskal-Wallis one-way analysis of variance.

^b^Chi-squared test.

At the post-delivery ultrasound, a total of 113 fibroids with a mean diameter of at least 10 mm were identified, while there was no evidence of the remaining 67 (37.2%) fibroids.

**Figure 2:**
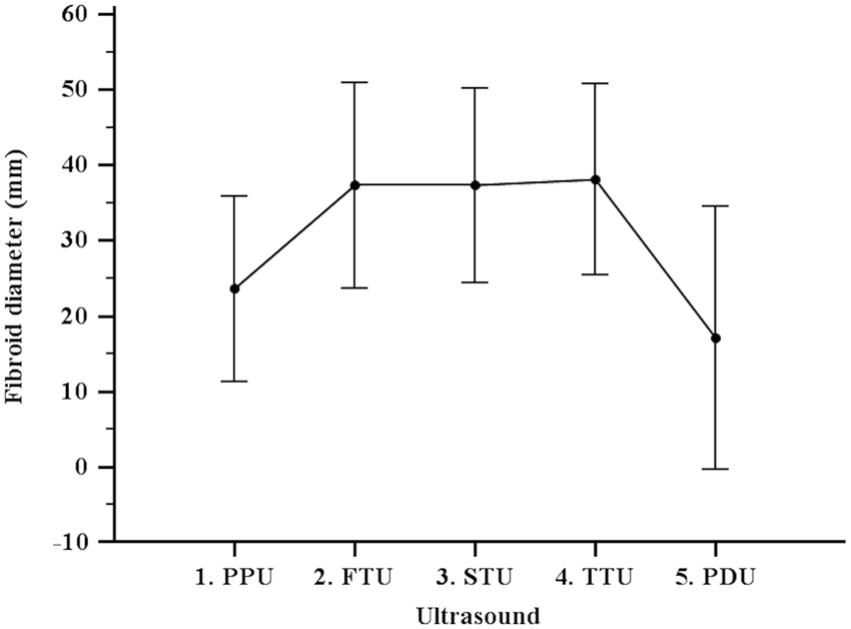
Comparison of fibroids diameter in the five ultrasounds.

A significant increase in median (IQR) diameter between the pre-pregnancy ultrasound (22 mm, IQR 15–30 mm) and the first-trimester ultrasound emerged (35 mm, IQR 28–43 mm), while the median (IQR) diameter of first-trimester ultrasound (35 mm, IQR 28–43 mm), second-trimester ultrasound (36 mm, IQR 30–42 mm), and third-trimester ultrasound (37 mm, IQR 30–43 mm), were not significantly different. A significant reduction in median (IQR) of post-delivery ultrasound (14 mm, IQR 0–30 mm), with respect to all previous ultrasounds, was noted. (Table 1, Fig. 2); this trend was also confirmed by the values of growth of fibroid diameter (GD%) and growth rate of fibroid diameter per week (GRw%) reported in Table 2.

**Table 2:** Growth of fibroid diameter % and growth rate per week between the five ultrasounds.

Characteristic	PPU - FTU (n = 180)	FTU - STU (n = 180)	STU - TTU (n = 180)	TTU - PDU (n = 180)	p
Growth of diameter (%)	83 (21–128)	0 (−5–8)	6 (0–9)	−60 (−100 – −31)	<0.001^a^
Growth rate week (%)	2 (1–4)	0 (0–1)	1 (0–1)	−1 (−2–0)	<0.001^a^

PPU = pre-pregnancy ultrasound; FTU = first-trimester ultrasound; STU = second-trimester ultrasound; TTU = third-trimester ultrasound; PDU = post-delivery ultrasound Data are reported as median (IQR).

^a^Kruskal-Wallis one-way analysis of variance.

According to the GD% between pre-pregnancy ultrasound and post-delivery ultrasound, 148 (82.2%) fibroids were “unchanged” or “regressed” fibroid diameter, and 32 (17.8%) fibroids presented an “increased” diameter.

In the bivariate analysis (Table 3), tobacco use, ongoing breastfeeding, a lower pre-pregnancy ultrasound diameter, and a higher GD% between pre-pregnancy ultrasound and first-trimester ultrasound were associated with an “unchanged or regressed” fibroid diameter with a p < 0.05.

**Table 3:** Comparison between “unchanged” or “regressed” fibroids (n = 148) and “increased” fibroids (n = 32) at the post-delivery ultrasound compared to the pre-pregnancy” ultrasound.

Characteristic	Unchanged or regressed (n = 148)	Increased (n = 32)	p
**Background**			
Age (yr)	34.6 ± 4.9	34.7 ± 3.1	0.91^a^
Body Mass Index	24.7 ± 3.7	24.3 ± 3.6	0.58 ^a^
Tobacco use	9 (6.8)	0 (—)	0.02^b^
No previous pregnancies	1 (0–1)	1 (0–2)	0.15^c^
Nulliparous	57 (38.9)	10 (31.3)	0.55^b^
Ongoing breastfeeding	90 (60.8)	10 (31.3)	<0.01^b^
**Obstetric outcomes**			
Gestational age at delivery (weeks)	39.0 ± 1.6	38.9 ± 1.6	0.71^a^
Preterm birth	12 (8.1)	3 (9.4)	0.91^b^
Vaginal birth	106 (71.6)	23 (71.9)	0.83^b^
**Sonographic**			
PPU diameter (mm)	20 (15–26)	30 (20–40)	<0.01^c^
FTU diameter (mm)	35 (28–40)	40 (30–49)	0.06^c^
STU diameter (mm)	35 (30–42)	40 (30–47)	0.19^c^
TTU diameter (mm)	37 (30–43)	38 (31–44)	0.72^c^
GD% PPU - FTU (%)	71 (28–150)	25 (0–72)	<0.01^c^
GD% FTU - STU (%)	1.3 (−3.8–8.2)	0 (−9.2–−4.7)	0.11^c^
GD% STU - TTU (%)	6 (3–8)	3.5 (−18–9)	0.13^c^

Data are reported as mean ± SD, median (IQR) or n (%) as appropriate.

^a^t-test.

^b^Chi-squared test.

^c^Mann-Whitney test.

In the multivariate model, only ongoing breastfeeding patients and a lower pre-pregnancy ultrasound diameter maintained an independent and significant association with a reduced or stable fibroid diameter, with an adjusted odds ratio (adOR) of 3.23 (95% CI; 1.35–7.70, p < 0.01) and 0.94 (95% CI; 0.91–0.97, p < 0.01), respectively.

Analyzing the percentage of “unchanged” or “regressed” fibroids at the post-delivery ultrasound according to ongoing breastfeeding, we found that the rate of “unchanged” or “regressed” fibroids was significantly higher in women with a pre-pregnancy fibroid diameter of 10–20 mm or 21–30 mm who were breastfeeding with respect of women who were not lactating, while there was no difference in case of higher pre-pregnancy diameters (Table 4).

**Table 4:** Comparison of “unchanged” and “regressed” fibroids at the post-delivery evaluation between lactating and non-lactating women, according to the four classed of pre-pregnancy fibroids diameter.

PPU diameter	Ongoing Breastfeeding	No breastfeeding	p
10–20 mm	51/54 (94.4)	26/34 (76.5)	0.03^a^
21–30 mm	25/26 (96.2)	7/27 (25.9)	<0.01^a^
31–40 mm	10/13 (76.9)	8/12 (66.7)	0.60^a^
>41 mm	4/7 (57.1)	3/7 (42.9)	0.45^a^

PPU = pre-pregnancy ultrasound.

Data are reported as n (%).

^a^Chi-squared test.

**Figure 3:**
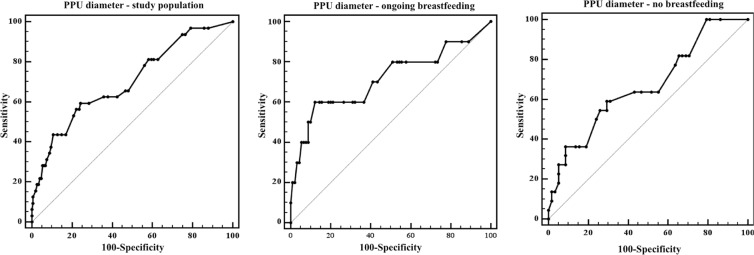
ROC curve analysis of pre-pregnancy ultrasound diameter with respect to the rate of “increased” fibroids in the study cohort, in ongoing breastfeeding subgroup, and no breastfeeding subgroup.

This relationship was confirmed by the Receiver Operating Characteristic (ROC) curve analysis of pre-pregnancy ultrasound diameter with respect to the rate of “increased” fibroids, which showed a significant cut-off of 32 mm of pre-pregnancy diameter for fibroid increase in dimensions in women who were breastfeeding, and a cut-off of 26 mm for women who were not lactating (Table 5, Fig. 3).

**Table 5:** ROC curve analysis of pre-pregnancy ultrasound diameter and “increase” in fibroid diameter at the post-delivery ultrasound with respect to ongoing breastfeeding.

	AUC	95% CI	Cut-off (mm)	Sensitivity	95% CI	Specificity	95% CI	p
Study population (n = 180)	0.7	0.6–0.8	>26	59.4	40.6–76.3	75.7	67.9–82.3	<0.01
Breastfeeding (n = 100)	0.7	0.6–0.8	>32	60.0	26.2–87.8	87.8	79.2–93.7	0.05
No breastfeeding (n = 80)	0.7	0.5–0.8	>26	59.1	36.4–79.3	70.7	57.3–81.9	0.02

## Discussion

In the present study, a significant growth of uterine fibroids in early pregnancy, a stabilization during the second half of pregnancy and a significant reduction in their dimensions in the post-partum period were observed. Indeed, fibroid diameter six months after delivery was significantly lower than the diameter measured during the pre-pregnancy evaluation and pregnancy evaluations, and 37.2% of fibroids were no more identified after pregnancy. Smaller fibroids were more likely to return to pre-gravidic dimensions or to regress in the post-partum period, particularly those with a pre-pregnancy diameter less than 26 mm. Ongoing breastfeeding seems to act as a protective factor against an increase in the size of uterine fibroids after pregnancy: women who were lactating six months after delivery were more likely to have an unchanged or regressed fibroid diameter (ad OR 3.23). The protective effect of breastfeeding seems to be more evident for fibroids with a pre-pregnancy diameter of less than 32 mm.

The negative trend in uterine fibroids dimensions after pregnancy is mostly consistent with existing literature, summarized by the recent systematic review by Vitagliano *et al*.^10^. Indeed, the authors report that adequate evidence supports fibroids growth during the first trimester, while contradictory evidence is available about the changes during the second trimester, third trimester, and puerperium^10^.

One of the most important limitations of previous studies is that the diagnosis of uterine fibroids was not made before pregnancy, with the exception of an observational study from Rosati *et al*.^17^ therefore, the global trend of fibroids dimensions may be obscured by the increase in dimensions that these lesions may undergo during early pregnancy^6^, so that the previously reported decrease in fibroid size could have been overestimated. However, in the present study, we have followed in each included patient the evolution of fibroids from pre-pregnancy to six months from delivery, noting a significant reduction in their diameter.

The mechanisms by which uterine fibroids may decrease in diameter or disappear in the post-partum period are not clearly understood yet, even if the mechanical and cellular changes that take place during birth and uterine involution can play a crucial role. Firstly, the transient uterine ischemia that occurs after delivery to avoid excessive blood loss after placental separation could have a direct effect on the survival of fibroid cells, that appear to be more sensitive to ischemic injury than normal myometrium. This effect on the survival of fibroid cells is in a way similar to the effects of laparoscopic bilateral occlusion of uterine arteries or uterine artery embolization^18–21^. The uterine involution after childbirth is reported to involve both mechanisms of apoptosis and proliferation, with an ultimate effect of remodeling of the existing tissues^22^. Apoptosis occurring during uterine involution could lead to selective loss of early neoplastic lesions^23,24^ and therefore, to a decrease in size or complete disappearance of small uterine fibroids^25^.

Among factors that could influence the involution process that seems to affect uterine fibroid in the post-partum period, we found that ongoing breastfeeding six months after delivery was positively associated with an “unchanged or regressed” fibroid diameter and this effect was particularly evident for fibroids with a pre-pregnancy diameter up to 32 mm.

This association could be explained by the period of suppressed ovarian steroid production, and the consequent decreased estrogen and progesterone exposure induced by lactation^26^. Previous epidemiologic studies have however shown mixed results regarding the association between breastfeeding and uterine fibroids: while Terry *et al*. reported in 2010 that duration of breastfeeding was inversely associated with fibroid risk^26^, other studies did not find any association^27,28^. The clinical study by Laughin *et al*. (2011)^29^ evidenced in 235 women with a diagnosis of uterine fibroids in early pregnancy that breastfeeding was not related to fibroid regression three-six months post-delivery. These conflicting results can be explained by the different dimensions of included fibroids, with probably a less evident effect of breastfeeding in case of large fibroids, and by the heterogeneous sensitivity of uterine fibroids to different mediators, not only represented by ovarian steroids, but also by other hormones^30^, mediators^31^, and growth factors^32^. In particular, Busnelli *et al*.^30^. indicate that oxytocin stimulates the proliferation of both myometrial and leiomyoma cells and could, therefore, counteract the effect of the hypoestrogenic state during breastfeeding on fibroid regression^29^. Mediators like Raf kinase inhibitor protein (RKIP) and growth factors activating multiple signaling pathways like Smad 2/3, ERK 1/2, PI3K, or β-catenin may contribute to regulate major cellular processes, including inflammation, proliferation, angiogenesis, and fibrosis which are linked to fibroids development and growth, both during pregnancy and in non-pregnant state^31,32^. It is possible to speculate that also the cellularity of fibroids^33^ may explain those different trends: the cellular component can grow or shrink in response to different hormonal levels, and the fibrous component could be more stable or be influenced by other non-hormonal mediators; however, further evidence is still needed in this regard. Furthermore, it is essential to emphasize that also epigenetic changes found in uterine fibroids, probably caused by some intrinsic abnormalities of the myometrium, abnormal myometrial receptors for estrogen or altered responses to ischemic damage may play a further role in fibroids growth and pathogenesis^34,35^.

None of the other considered factors was significantly associated with post-partum fibroid dimension and, in particular, no difference was found between women who delivered vaginally or by cesarean section, in line with previous literature^29^.

Although we did not find a high rate of cesarean section in our cohort, this may represent a severe pregnancy complication in women with uterine fibroids^36^. Cesarean section may lead to both short-term and long-term health effects for women and children^37,38^, as well as implications for future pregnancies^39^. Therefore, specific prevention procedures should be adopted, with accurate identification of women that could benefit from this intervention rather than be selected for a vaginal birth.

The strengths of the present study are a large number of included patients and that only women with a pre-pregnancy diagnosis of uterine fibroids who have subsequently become pregnant were included and accurately monitored during pregnancy and after delivery. Sonography is currently the diagnostic method of choice for fibroids but includes an inevitable degree of inaccuracy that might compromise the accuracy of size determination. However, the same experienced sonographer performed all the evaluation, with a standardized method of measurement. Moreover, the ultrasonographic differential diagnosis between fibroids and uterine sarcomas remains a challenge, and there is no clear consensus on the ultrasound features of uterine sarcoma. Recent evidence reports that in case of solid uterine masses with inhomogeneous echogenicity, irregular cystic areas, absence of fan-shaped shadowing, and moderately to very well vascularization^40^, further evaluation and appropriate management are needed^41^, considering the poor prognosis and the therapeutic possibilities^42^.

In conclusion, the impact of pregnancy on uterine fibroids seems to be limited to pregnancy itself. Indeed, after an increase in size during early pregnancy, uterine fibroids tend to stabilize and undergo a regression process that may start in the second-third trimester but is accelerated by events of childbirth and post-partum. Clinicians need to be aware of such trend, in order to correctly inform and manage patients with uterine fibroids who become pregnant, expecting an early growth, with a possible risk of adverse obstetric outcomes^14^ or fibroid-related complications^43^, but also a return to a pre-gravidic “steady-state”, or even a reduction or resolution of such lesions. The involution process of the uterus and hormonal fluctuations during puerperium seems to influence this trend. Breastfeeding could have an additional role in the regression of uterine fibroid after birth. The positive effect of breastfeeding seems to be more evident in fibroids with a diameter up to 32 mm, and in these women, a return to a pre-gravidic state, a reduction or a regression of such lesions seems to be more likely.

## Methods

This was a monocenter observational study carried on child-bearing age patient with a diagnosis of uterine fibroids performed at the gynecological ultrasound unit of our institution from January 2007 to December 2016, with no indications for immediate treatment, who had a pregnancy within one year from diagnosis, and who were re-evaluated six months after delivery. Women were initially eligible if they were diagnosed with at least one fibroid with a mean diameter greater than 10 mm before pregnancy, identified during a transvaginal ultrasound performed for mild gynecological symptoms (abnormal uterine bleeding, pelvic pain, and urinary tract or bowel compression) with no immediate need for medical or surgical treatment

Exclusion criteria were the presence of more than four fibroids, the evidence of submucosal fibroids, the suspicion of adenomyosis, and history of previous medical or surgical treatment for fibroids. Women with more than four fibroids were excluded because of the difficulty to perform the complete ultrasounds assessments accurately. All women with a spontaneous singleton pregnancy who occurred within one year from the initial diagnosis of fibroids and who performed the six months’ post-delivery ultrasound evaluation were considered for the final analysis. Patients subjected to *in-vitro* fertilization, with multiple pregnancies, diagnosed with miscarriage or ectopic pregnancy, or using hormonal contraceptives in the post-delivery period were excluded. All patients enrolled in the present study underwent routine pregnancy and post-delivery assessments and delivered at our center. Data were obtained by clinical charts, and each woman signed informed consent to ultrasound execution and data collection. All procedures performed were in accordance with the ethical standards of our institution and with the 1964 Helsinki declaration and its later amendments. The study protocol was approved by local ethical committee (Comitato Etico Regionale Marche).

All included women underwent a total of five ultrasound examinations, the first in the pre-pregnancy period within one year before the last menstrual period (pre-pregnancy ultrasound), the second between 10 and 13 complete gestational weeks (first-trimester ultrasound), the third between 19 and 21 complete gestational weeks (second-trimester ultrasound), the fourth between 30 and 32 complete gestational weeks (third-trimester ultrasound), and the fifth six months after delivery (post-delivery ultrasound).

Ultrasounds were performed with a Voluson 730 PRO (General Electric Healthcare, Milwaukee, WI, USA) and a 3.5–5.5 MHz probe by the same senior sonographer.

Fibroids were defined as well-defined round uterine lesions, with heterogeneous echogenicity, shadows at the edge of the lesion or internal fan-shaped shadowing, and circumferential flow around the lesion. Three perpendiculars diameters (D1, D2 and D3 in mm), the location (anterior, posterior, right lateral, left lateral or fundal) and the site (subserosal or intramural) were collected for each fibroid, as previously published^44^.

The background characteristics considered were: maternal age at pregnancy, BMI, smoking, number of previous pregnancies, nulliparity, and ongoing breastfeeding at the ultrasound evaluation six months after delivery, defined as suckling or pumping at least four times per day^29^. Pre-pregnancy maternal height and weight were measured, and the body mass index was calculated.

Obstetric outcomes considered included: gestational age at delivery, rate of preterm birth (less than 37 complete gestational weeks), and mode of delivery (vaginal birth or cesarean section).

The statistical software used was SPSS 20 (SPSS Inc., Chicago, IL, USA). The normality of each variable was evaluated using the D’Agostino- Pearson test. Variables with normal distribution (age, body mass index, and gestational age at delivery) were expressed as arithmetic mean ± standard deviation, while skewed variables (number of previous pregnancies and fibroid diameter) were reported as median and interquartile range (IQR). Qualitative variables were expressed as a percentage.

The comparison of fibroid diameter between all the ultrasound evaluations was accomplished using the Kruskal-Wallis one-way analysis of variance. A p < 0.05 was regarded as statistically significant.

For all the following fibroid diameter determinations, we considered the largest of the three measured perpendicular diameters for each fibroid. We defined as “change in fibroid diameter” between two ultrasounds the difference between the last diameter of each fibroid and the starting fibroid diameter. The following formula defined growth of fibroid diameter (GD%): GD% = (100* change in fibroid diameter/starting fibroid diameter), whereas growth rate of fibroid diameter per week (GRw%) was calculated for each fibroid using the formula: GRw% = GD%/the interval in weeks between the periods considered^7,14^. The GD% and the GRw% were calculated for the interval between each ultrasound.

We also determined the GD% between pre-pregnancy ultrasound and post-delivery ultrasound, defining a fibroid as “increased” if GD% was above 40%, as “unchanged” if GD% was between 40% and −40%, and “regressed” if GD% was less than −40% or if there was no evidence of fibroids at the post-delivery ultrasound. In order to identify the possible factors associated with the increase in fibroids diameter between the pre-gravidic and the post-partum period, we performed a bivariate analysis comparing patients with an “unchanged” or “regressed” fibroid diameter and patients with an “increased” fibroid diameter. All the variables that in the bivariate analysis presented a p < 0.05 were included in a multivariate logistic model as independent variables, and the rate of “unchanged” or “regressed” fibroids as a dependent variable. In order to evaluate the association between pre-pregnancy diameter, ongoing breastfeeding, and the dimensional variations of fibroids in the post-partum period, we compared the rate of “unchanged” or “regressed” fibroids between women who were breastfeeding and women who were not lactating for the following classes of fibroid pre-pregnancy diameter: 10–20 mm, 21–30 mm, 31–40 mm and ≥40 mm. Subsequently, we performed a Receiver Operating Characteristic (ROC) curve analysis to identify a cut-off of pre-pregnancy diameter predictive for post-partum fibroid diameter “increase”.

## Data Availability

The datasets analysed during the current study are available from the corresponding author on reasonable request.
